# A New Design Scheme for Intelligent Upper Limb Rehabilitation Training Robot

**DOI:** 10.3390/ijerph17082948

**Published:** 2020-04-24

**Authors:** Yating Zhao, Changyong Liang, Zuozuo Gu, Yunjun Zheng, Qilin Wu

**Affiliations:** 1 School of Management, Hefei University of Technology, Hefei 230009, China; 2012010126@mail.hfut.edu.cn (Y.Z.); cyliang@163.com (C.L.); 2 School of Economics and Management, Hefei Normal University, Hefei 230601, China; 3 Department of Art Design, Anhui University of Arts, Hefei 231635, China; zzgu0551@163.com; 4 Anhui Key Laboratory of Digital Design and Manufacturing, Hefei University of Technology, Hefei 230009, China; 2017110093@mail.hfut.edu.cn

**Keywords:** upper limb rehabilitation robot, natural interaction, cooperative communion, control

## Abstract

In view of the urgent need for intelligent rehabilitation equipment for some disabled people, an intelligent, upper limb rehabilitation training robot is designed by applying the theories of artificial intelligence, information, control, human-machine engineering, and more. A new robot structure is proposed that combines the use of a flexible rope with an exoskeleton. By introducing environmentally intelligent ergonomics, combined with virtual reality, multi-channel information fusion interaction technology and big-data analysis, a collaborative, efficient, and intelligent remote rehabilitation system based on a human’s natural response and other related big-data information is constructed. For the multi-degree of the freedom robot system, optimal adaptive robust control design is introduced based on Udwdia-Kalaba theory and fuzzy set theory. The new equipment will help doctors and medical institutions to optimize both rehabilitation programs and their management, so that patients are more comfortable, safer, and more active in their rehabilitation training in order to obtain better rehabilitation results.

## 1. Introduction

With the increasing populations of the disabled and the elderly, scientific and technological innovation to maintain and improve the health of members of these populations has become a strategic need in many countries [1,2]. The research and development of advanced rehabilitation robots to achieve functional compensation and reconstruction of patients with dysfunction or a lack of function is socially significant. It will promote the development of elderly health services and public welfare undertakings for the disabled. In 2015, the global market share of rehabilitation robots was $577 million USD. It is estimated that, by 2020, this number will reach $1.73 billion USD [3]. This very high growth rate is unmatched by other industries.

A rehabilitation robot is a kind of automatically operated machine that is designed to improve movement in persons with impaired physical functioning [4]. The rehabilitation robot was first designed in the 1980s [5]. After 1990, the research on these robots developed more rapidly. In terms of the robot mechanism, MIT-Manus [6,7,8,9] is one of the earliest robots developed for rehabilitation. Loureiro et al. established the GENTLE/s system based on tactile and virtual reality visualization technology in 2003 [10]. Hesse et al. developed the training robot Bi-Manu-Track [11], which has one degree of freedom and can perform passive and active exercises of bilateral forearm pronation/supination, wrist flexion, and wrist extension. A rehabilitation trainer ARM Guide [12] was developed by the University of California and the Rehabilitation Research Institute of Chicago, which has three controllable degrees of freedom. It can reach out in a straight direction using a motor, and can deflect and lift a shaft using a magnetic powder brake. The University of Washington developed an upper limb exoskeleton rehabilitation robot named CADEN-7 [13], which uses the rope driving mechanism to separate the motor and the joint in order to minimize the wear on the part resembling the upper arm of the human body. The arm rehabilitation robot named ARMin [14], developed by the University of Zurich, Switzerland, consists of four active and two passive degrees of freedom. Each joint is equipped with position and force sensors, which can assist in some clinical sports training. Arizona State University developed a multi-degree-of-freedom, upper-limb-assisted training robot, RUPERT [15], which uses an exoskeleton-like wearable structure. Yu et al. of the National University of Singapore designed a flexible actuator as a series elastic actuator of a rehabilitation robot arm [16,17]. Xu et al. developed a variable resistance pneumatic robot system for bilateral, upper limb interactive training [18]. Bolboacă et al. of Luliu Hațieganu University of Medicine and Pharmacy considered ethical issues when designing exoskeleton devices for patients [19]. There are also other types of a rehabilitation robot. Dovat et al. designed a cable-actuated rehabilitation system in which each finger is attached to an instrumented cable loop, which allows force control and a predominantly linear displacement [20]. Mao et al. designed a Cable-Driven Arm Exoskeleton (CAREX) for neural rehabilitation to achieve desired forces on the hand in any direction, as required in neural training [21]. Chen et al. proposed a cable-driven parallel waist rehabilitation robot, which can accurately implement the relative lateral bending, flexion, extension, and rotation of the waist on the premise of the safety, to assist the patients with waist injuries to do some rehabilitation training [22]. Chen et al. designed a two-degrees-of-freedom tethered exoskeleton that can provide independent torque control on elbow flexion/extension and forearm supination/pronation by two identical series elastic actuators (SEAs), which are coupled through a novel cable-driven differential [23].

In terms of the control method and dynamic modeling, Hogan [24] first proposed the impedance control method, which can be used to achieve compliance control of the robot. Richardson [25] developed a three-degree-of-freedom upper limb rehabilitation robot for physical therapy, and applied a linear optimization strategy in the robot mechanism. Rahman [26,27] merged the nonlinear, sliding-mode control with an exponential approach law, which takes into account the dynamic modeling of the robot system and the nonlinear motion of the upper limb. This method greatly reduces the chatter and improves the dynamic tracking performance, but the calculation of the controller requires high modeling accuracy. Jinwu’s team at the Shanghai Jiaotong University designed a robust controller for the two-degree-of-freedom, upper limb rehabilitation robot, which makes the robot system robust and causes the tracking error to tend to zero under certain conditions with a good control effect. However, the high tracking accuracy is achieved by sacrificing the large control input torque [28]. Aiguo’s team at the Southeast University in China applied an adaptive impedance controller based on a fuzzy neural network to an upper limb rehabilitation robot. This setup can adjust the action between the robot and the damaged limb in real time, according to the recovery of the damaged limb. However, these dynamic control methods do not consider the uncertainty, transient stability, and structural constraints of the upper limb rehabilitation robot [29]. Based on the upper limb rehabilitation exoskeleton robot system, researchers at the Nanjing University of Aeronautics and Astronautics proposed a fuzzy sliding-mode admittance control strategy to realize a training process controlled by the coordination of a human and machine [30]. Culmer et al. proposed a cooperative control scheme used by the intelligent pneumatic arm movement (iPAM) system to deliver safe, therapeutic treatment of the upper limb during voluntary reaching exercises [31]. Xu et al. designed a novel hybrid control algorithm for upper-limb rehabilitation with a fuzzy-logic based PID (Proportion Integral Derivative) position control strategy and a fuzzy adaptive impedance force controller [32]. Zhang et al. presented a theoretical framework that established the passivity of the closed-loop upper-limb rehabilitative robotic systems and allowed rigorous stability analysis of human-robot interaction [33]. Riani et al. designed a robust adaptive integral terminal sliding mode control strategy to deal with unknown but bounded dynamic uncertainties of an upper limb exoskeleton system in order to achieve passive rehabilitation movements [34].

In fact, robotic devices are already used in clinical practice as well as in clinical evaluation. Some of them have succeeded to target the subject group such as Kiguchi’s [35], Cheng’s [36], Cozens [37], Mavroidis [38], and more [39]. Some limitations for these robots still exist, including joint space limitations, shortages in mechanism flexibility, user-unfriendly interactive training, unsuitability for intelligent remote management, and other key technical issues. Therefore, it is urgent that researchers study and develop a more flexible, intelligent, and safe rehabilitation robot.

## 2. Design of the Rehabilitation Robot Structure

**Figure 1:**
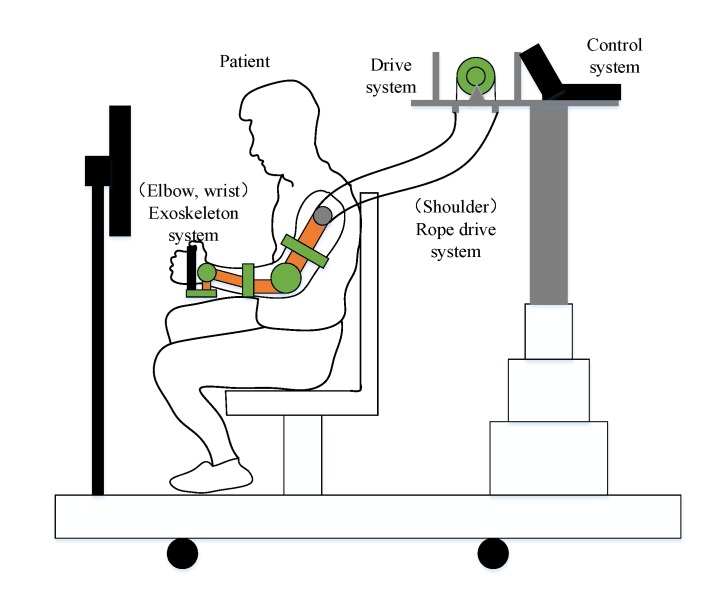
Overall design of the structure of the rope-driven exoskeleton multiplexing robot.

In traditional rehabilitation robots, a driving motor is placed at every joint of the exoskeleton, which leads to a large number of connections with the human body, and seriously affects the motion characteristics of the rehabilitation robot. Combining the rope-driven robot with the exoskeleton robot, the advantages of both can be used, in terms of the characteristics of force application and motion output of the human body in ergonomics, especially the force of arm control and the design criteria of relevant motion mechanisms [40]. A rope is used to drive each moving joint, so that the driving motor is placed outside the connecting part of the human body, and the mass of the moving mechanism is reduced. The other part of the robot, known as the exoskeleton structure, ensures strength and accuracy. In the overall design process, the following basic requirements must be met. The exoskeleton joints and human joints must be accurately matched, and the length of the exoskeleton robot must match the length of the human upper limbs. The basic structure is shown in Figure 1.

In the design of the exoskeleton of the manipulator and wrist, starting from the human body structure, the rotation axis of each joint should be in the same line with the rotation axis of the human body joint. The shoulder joint may be approximated as a ball joint, but it is impossible to achieve the intersection of three degrees of rotational freedom of the shoulder joint in the interior of the shoulder joint at present. In this paper, the exoskeleton is driven by the flexible rope to assist the shoulder joint of the human upper limb in using three degrees of freedom of movement in order to achieve the desired flexibility. The elbow and the wrist each have two degrees of freedom. The exoskeleton is used to achieve the four degrees of freedom of joint movement in order to ensure the full freedom of movement during rehabilitation of the upper limbs. The targeted motions for the rehabilitation device are determined according to different injury causes, different patients, and different needs of different rehabilitation training periods.

**Figure 2:**
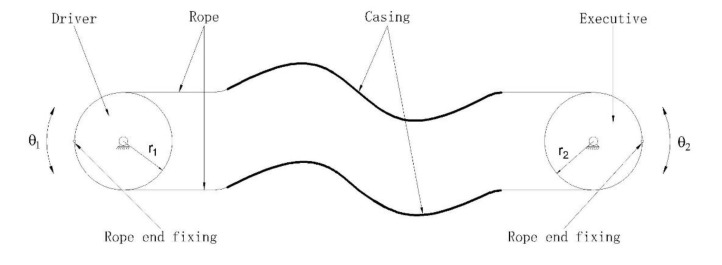
The double-lasso drive mode.

**Figure 3:**
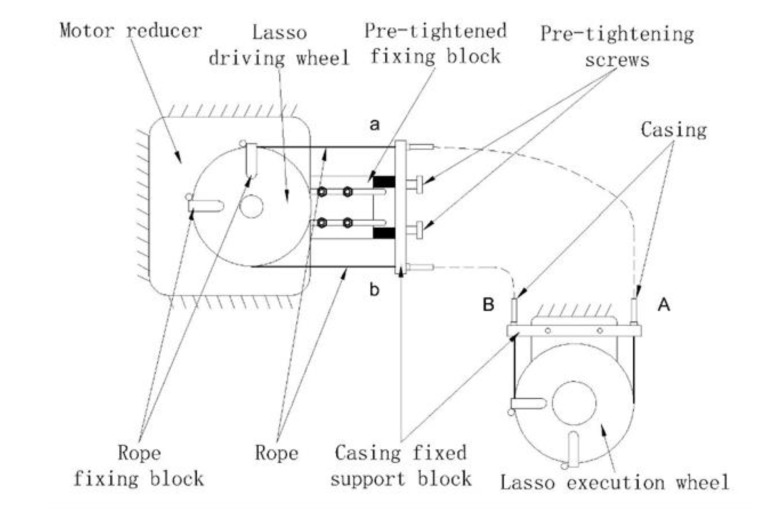
Schematic diagram of the double-lasso drive system.

The rope-driven system is generally composed of casing and rope with relative motion, which has the advantages of a simple structure, flexibility, and the long-distance transmission of power. There are two common methods of rope transmission, single-lasso transmission, and double-lasso transmission. The single-lasso drive is generally used to achieve linear reciprocating motion of the mechanism. The drive end can only provide one direction of rotation, and rotation in the other direction is generally provided by a spring and other elastic elements. The double-lasso drive is used to achieve rotary movement of the mechanism, and the drive end can provide two directions of rotation. The joint of a shoulder joint rehabilitation device is a rotary joint. Therefore, the double-lasso transmission mode is adopted. The lasso transmission mode is shown in Figure 2. The double-lasso drive system includes the lasso driver and parts to pre-tighten the lasso. The principle is shown in Figure 3. Table 1 gives the comparison of some robotic devices for upper limb rehabilitation.

## 3. Design of the Human-Machine Natural Interaction Scheme Based on Perceived Information

A goal of the research is to enable doctors and patients to better interact with robots and make the rehabilitation process better, safer, and easier to accept, based on the basic principles of human-machine interaction (i.e., user control, intuitiveness, observability, ease of use, timely response, simplicity, and consistency [45]), using modern sensing technology, identification technology, information technology, and more. In light of this, a new multi-sensor human-machine natural interaction method is proposed, which uses a multi-sensor system to collect information about the external environment, and which uses multi-sensor fusion processing technology to comprehensively process all kinds of perceived information. This interaction method is applied to the rope-traction upper limb rehabilitation robot system to improve the robot’s performance and the human-machine interaction.

### 3.1. Method to Process and Coordinate Multiple Pieces of Information

We first built the external sensor system of the robot, which can perceive the patients, doctors, and rehabilitation environment, and obtain relevant information. The sensors involved include a stereo vision sensor, auditory sensor, force sensor, proximity sensor, and electromyographic (EMG) sensor. The stereo vision sensor collects and recognizes the patient’s actions and expressions. The auditory sensor collects and recognizes the patient’s and doctor’s language. The force sensor detects the force of pulling on the rope. The proximity sensor detects obstacles in the rehabilitation process and the EMG sensor collects the EMG signal of the patient during the rehabilitation process. The construction of a multi-sensor system can reduce the shortcomings of using a single sensor, such as having limited or incomplete information and uncertainty, and can describe the human-machine environment of the robot comprehensively.

Each piece of information is different in space, time, expression, and purpose. Therefore, a multi-information processing method is needed for information processing and management to coordinate the work of each sensor with each other sensor and deal with all kinds of information of the multi-sensor system more effectively. This paper constructs the following processing method. First, the information collected by the external sensors of the robot is classified with the classification corresponding to the form of the information, which can be divided into redundant information and complementary information. Redundant information can improve system fault tolerance and reduce system uncertainty. Complementary information can improve the integrity and correctness of the system description environment. Second, the coordinated management of multi-sensor information is achieved by sensor selection, coordinate transformation, data transformation, and using a sensor model database. Lastly, a method of fusing the information based on parameter estimation is adopted by the information fusion processor to coordinate the management and fusion of the multi-sensor information.

### 3.2. Human-Machine Natural Interaction Method Based on the Multi-Sensor System

**Figure 4:**
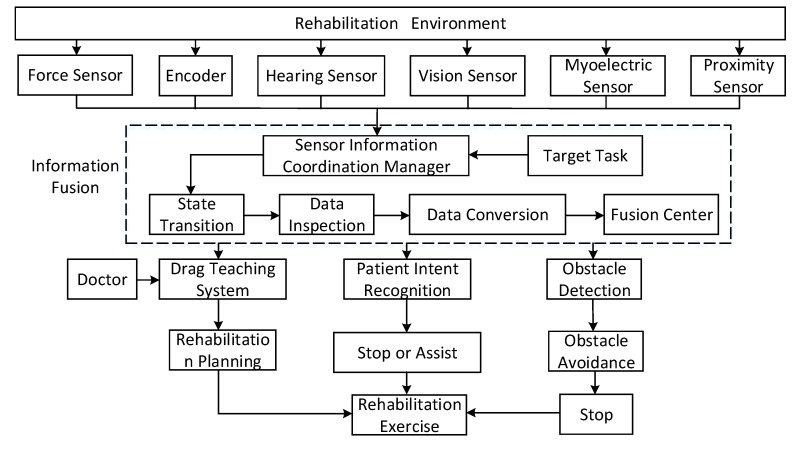
A human-machine natural interaction method based on the multi-sensor system and multi-information fusion method.

**Figure 5:**
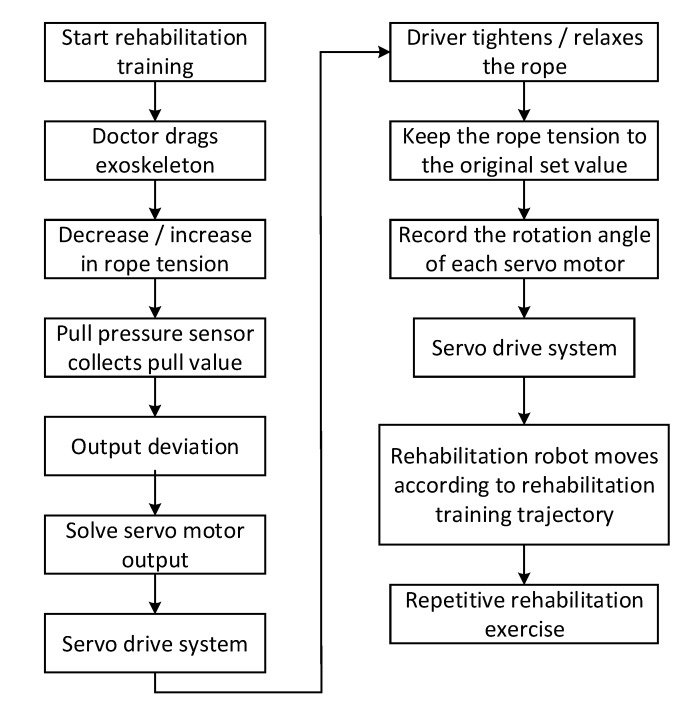
The dragging teaching system.

A human-machine natural interaction method based on the multi-sensor system and coordinated information is designed based on actual needs of patients and existing technologies, as shown in Figure 4. The measured value of the force of pulling the rope and the position information of the driving motor are collected by the robot’s multi-sensor system, and, through the multi-information fusion processing technology, a set of dragging teaching systems for the robot is designed. This is shown in Figure 5. As a human-machine natural interaction method, the dragging teaching system can effectively simplify the programming process of the rehabilitation training trajectory planned by doctors and improve the rehabilitation training effect on patients. In addition, through the robot’s multi-sensor system, which collects the patient’s motion, language, EMG (Electromyogram), and other sensory information, and through the multi-information fusion processing technology, which identifies the intention of the patient. The robot can intelligently assist the patient in carrying out the rehabilitation training and ensure the patient’s safety during training.

## 4. Design of the Scheme to Integrate Human, Machine, and Environment Based on a Human’s Natural Response

It is important to construct a human-machine cooperative rehabilitation system for the rehabilitation robot, patient, doctor, and environment. Therefore, the theory and principles of machine learning and deep learning are applied to the rehabilitation training platform, which is human-friendly, machine-friendly, and environmentally-friendly [46,47]. A multi-dimensional rehabilitation system based on a human’s natural response and other, related big-data information is constructed. The system is built by combining ergonomics, virtual reality, multi-channel information fusion interaction technology, big-data analysis, and deep-learning theory.

### 4.1. Rehabilitation Training Platform with Friendly Cooperation among Human, Machine, and Environmental Elements

**Figure 6:**
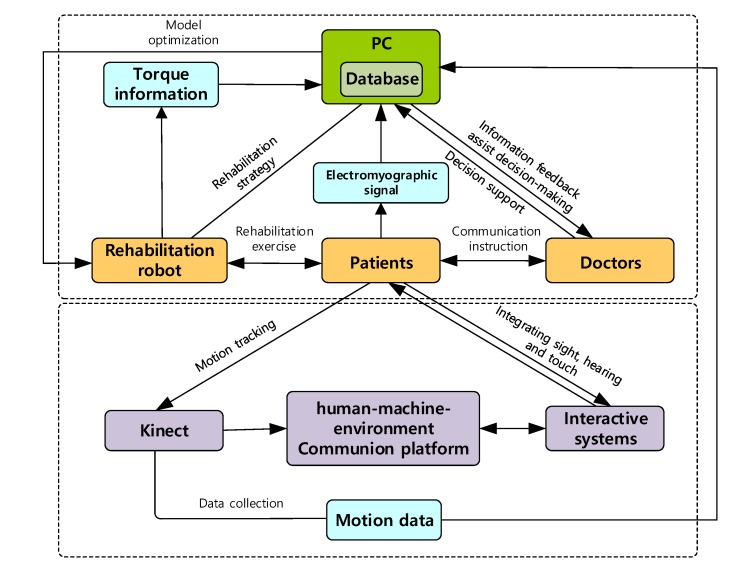
Kinect-based intelligent upper limb rehabilitation system.

In view of the shortcomings of existing upper limb rehabilitation robots, such as the lack of autonomy, adaptability, and intelligence, the limitations of the human-machine interaction, and the single data connection, which all result in a lack of collaborative control among multiple platforms when ergonomics are introduced to the robot. The natural interaction, accessibility, cognitive needs, cultural factors, and aesthetics are comprehensively considered for four factors: the rehabilitation robot, patient, doctor, and environment. At the same time, the multi-channel integration of visual interaction, speech recognition, gesture input, tactile feedback, and other interactive information technologies are combined with virtual reality technology to build a Kinect-based upper limb rehabilitation training interaction system, and form a rehabilitation training platform with friendly collaboration among humans, machines, and environmental elements, as shown in Figure 6.

The Kinect-based upper limb rehabilitation auxiliary equipment is a multi-coupling system that integrates action capture, information interaction, and virtual reality, and can design corresponding rehabilitation training control strategies, according to the clinical characteristics of patients in different rehabilitation stages. In addition, the interactive software system based on virtual reality technology integrates a variety of interesting games, which supports relevant rehabilitation training courses, and even simulates real-life activities such as cooking, cleaning, and playing chess to improve patients’ initiative to participate in rehabilitation training and achieve better rehabilitation training results.

### 4.2. Interactive Training System Based on a Human’s Natural Response and Other Related Data

On the basis of the upper limb rehabilitation equipment equipped with multi-intelligent sensors, a hierarchical progressive rehabilitation training mode (including passive mode, semi-passive semi-active mode, and active mode) is designed according to the different rehabilitation stages and different rehabilitation needs of patients.

**Figure 7:**
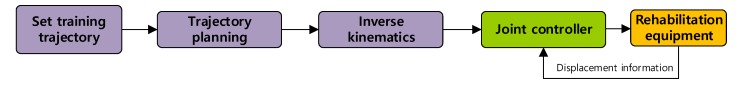
System control diagram of the passive training mode.

In the early stage of rehabilitation, the fuzzy sliding-mode variable-structure control algorithm is used. Based on the preset path, the upper limb rehabilitation training robot pulls the affected limb for passive-mode training. The system diagram is shown in Figure 7.

**Figure 8:**
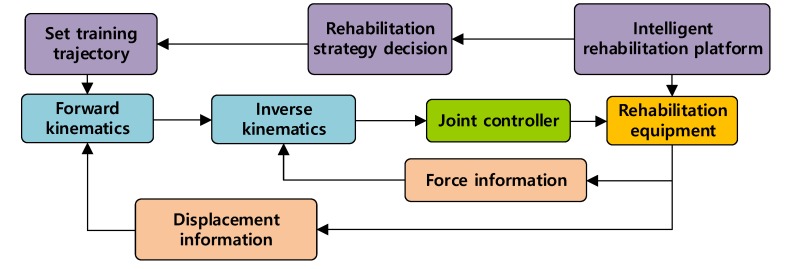
System control diagram of the semi-passive, semi-active training mode.

The admittance control algorithm based on the minimum interference principle is adopted for patients in the middle stage of rehabilitation. According to the deviation in movement of the affected limb, the control area is divided, and the auxiliary force provided by the robot is adjusted to help the patient complete the semi-passive-mode and semi-active-mode training. The system diagram is shown in Figure 8.

**Figure 9:**
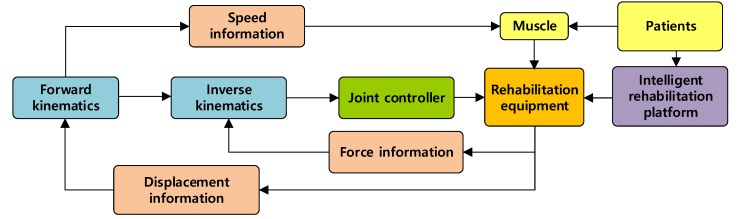
System control diagram of the active training mode.

An active rehabilitation training strategy based on admittance control and virtual reality games is adopted for patients in the later stage of rehabilitation to enhance interaction with and interest in the training process. The patients resist the force imposed during active training treatment, as shown in Figure 9.

When patients participate in rehabilitation training and immerse themselves in rehabilitation games, multi-modal information such as EMG signals, kinematics signals, and dynamics signals are collected continuously. Since the EMG signal has a high correlation with the force produced by muscle contraction, and affects the patient’s final movement, the force or moment sensor signal can accurately and reliably reflect the movement information of the upper limb. Therefore, by extracting features from the collected signals, the patient’s intended motion can be recognized.

Through intelligent sensors and Kinect, a large amount of multi-modal information such as EMG signals, kinematics signals, and dynamic signals related to the natural responses of the human body, such as responses related to vision, hearing, and touch, are collected. Based on the characteristics of the movement task, methods such as neural network methods, big-data analysis, and deep learning are used to achieve the accurate modeling and prediction of the individual movement mode of the patient, and to continuously improve recognition of the human’s intended motion. Thus, the interactive training system based on the natural responses of the human body and other related big-data information can be continuously improved.

### 4.3. Multi-Dimensional Intelligent Rehabilitation System

Through the effective collection and arrangement of the multi-platform and multi-system rehabilitation data of different patients, doctors, and rehabilitation robots, a multi-dimensional rehabilitation training and evaluation database can be established. A three-layer intelligent rehabilitation platform based on data collection, data analysis, and data service can be built by using the theory of big-data analysis and deep learning. Thus, a multi-level linkage rehabilitation system is formed to maximize the use of rehabilitation information resources.

**Figure 10:**
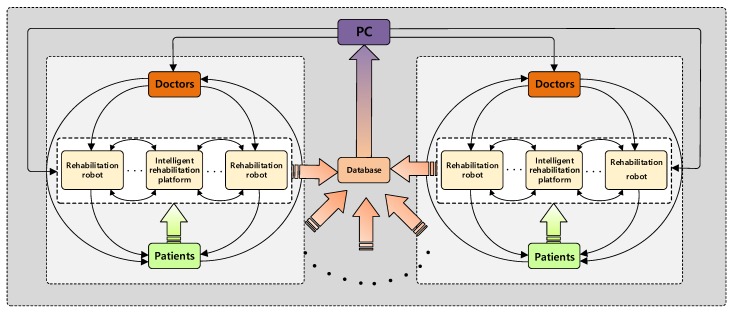
Multi-dimensional intelligent rehabilitation system.

First, according to the scientific quantitative evaluation method, which truly reflects the sports function, a quantitative evaluation table is made and a personalized training scene is designed. Then, according to different training scenarios, a scientific, quantitative evaluation method is developed based on big data, and a comprehensive evaluation model of rehabilitation is established based on AHP (Analytic Hierarchy Process). Lastly, a multi-dimensional intelligent rehabilitation system guided by data analysis results is formed to assist doctors in decision-making. The multi-dimensional intelligent rehabilitation system is shown in Figure 10. We extract knowledge from the big data of historical rehabilitation training and build a case knowledge-based system founded on rehabilitation big data. According to the real-time data of physical and mental health of each rehabilitation personnel, the personalized rehabilitation training scheme is matched from the historical case knowledge base through a deep neural network. Moreover, the training scheme is adjusted and optimized dynamically [48,49].

## 5. Dynamic Modeling and Control Scheme Design

### 5.1. Dynamic Modeling

**Figure 11:**
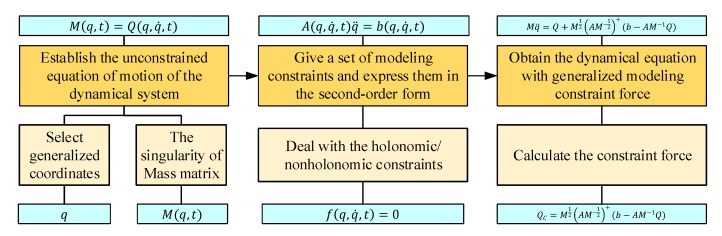
The three-step Udwadia-Kalaba modeling method.

The dynamic model of the rehabilitation robot is very important for the design of the system controller, which is related to the control accuracy and other important performance indexes of the system. For this kind of complex, multi-body mechanical system, especially a multi-body system with a closed-loop structure, the existing mechanical system dynamic modeling methods, such as the Newton-Euler method and Lagrange method, are difficult to use for modeling and for obtaining the analytical dynamic equations. That is because the Lagrange multiplier method relies on problem-specific approaches to determine the multipliers and it is often very difficult to find the multipliers to obtain the explicit equations of motion for systems that have large numbers of degrees of freedom and a mass of non-integrable constraints. The Udwadia-Kalaba dynamic theory [50,51,52,53,54] has been proposed in recent years and provides a new modeling method for multi-body mechanical systems. The innovation of this method is mainly in the hierarchical clustering of subsystems. The motion equations of the system obtained by this method are expressed analytically without additional physical variables. The segmentation and aggregation operations involved in the system modeling process can be implemented in multiple levels, and each constraint can be added, removed, or modified at any time. Compared with the existing dynamic modeling methods, this method effectively simplifies the dynamic analytical modeling of complex multi-body systems, as shown in Figure 11.

There are three steps to establish the dynamic model of the rope-traction and exoskeleton-multiplexing upper limb rehabilitation training robot using the dynamics theory of Udwadia-Kalaba. First, for the rope traction and exoskeleton, the unconstrained motion equations of each subsystem are obtained by the Newton-Euler or Lagrange method, and then the unconstrained motion equations in matrix form are obtained. It should be noted, however, while Newton-Euler or Lagrange method is not easily applied to a multi-body system, it is available for each subsystem of the whole system. Second, all the motion constraints of the system are written in second-order form, and the second-order structural passive constraint equation in matrix form is obtained. Lastly, the constraint force is obtained based on the Udwadia-Kalaba equation and added to the unconstrained motion equation. These steps result in the dynamic equations of the robot.

### 5.2. Controller Design

**Figure 12:**
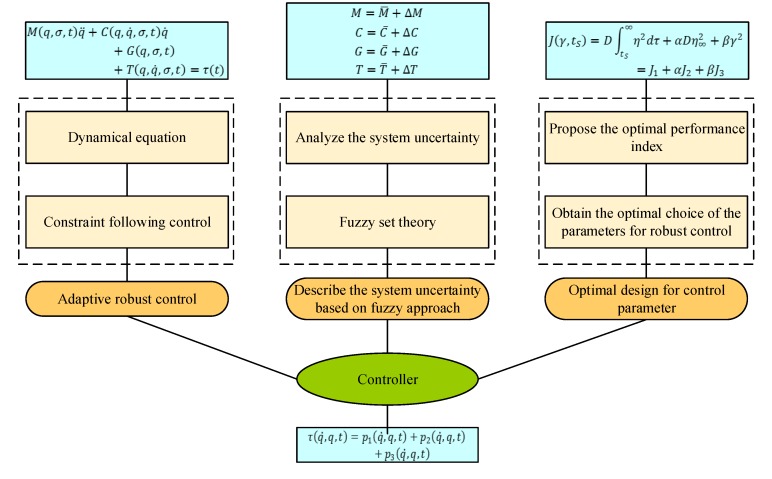
Optimal, adaptive, robust control design.

With the dynamic model, the control system can be designed. Because of the high nonlinearity of the robot and the uncertainty of some control parameters, controller design is generally difficult. Therefore, an optimal, adaptive robust control theory based on fuzzy set theory is proposed, as shown in Figure 12. First, the model of uncertain parameters based on a fuzzy set is established, according to the analytic dynamic model. Second, the performance index of the fuzzy system is established. Lastly, the feedback gain of the controller is optimized based on the fuzzy information. Thus, the deterministic, adaptive robust control based on fuzzy theory is designed, which can make the uncertain, complex multi-rigid body mechanism reach actual stability.

**Figure 13:**
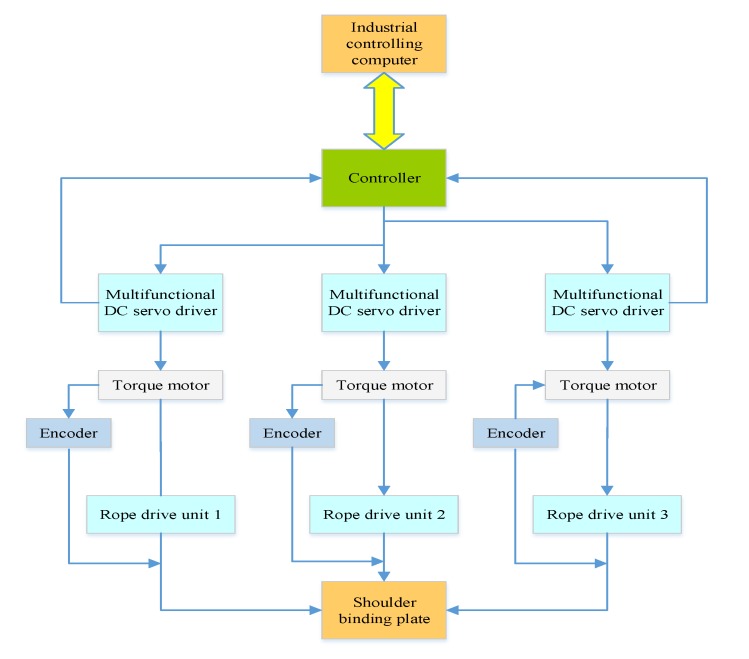
Control structure diagram of the rope traction part of the system.

In the system, the rope traction part of the system is a redundant driving system, which increases the difficulty of robot compliance control. Therefore, an impedance control strategy based on the inner force loop and the outer position loop is adopted, and the position of the shoulder joint node is controlled by the driving forces and positions of rope 1, rope 2, and rope 3. The control structure diagram is shown in Figure 13. The control process of the robot control system is as follows. First, the force between the robot and the external environment is detected by the tension sensor. Then, the driving force value of each rope is calculated by using the optimization model. Third, the results are applied to the impedance control system of the inner force loop and the outer position loop. Lastly, the active compliance control of the upper limb rehabilitation robot is achieved.

**Figure 14:**
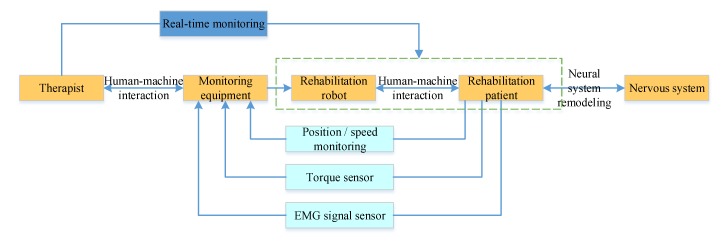
Control system diagram of the exoskeleton.

Now, according to the design principles and requirements of the system, the exoskeleton control system of the upper limb rehabilitation robot can be completed, as shown in Figure 14. The system integrates multi-sensor information, which can evaluate the rehabilitation status, according to the real-time movement data of patients, select the corresponding training plan, and set the robot’s task. The robot receives and performs tasks, and drives patients’ affected limbs to carry out different modes and intensities of sports training, in order to achieve targeted rehabilitation training for each patient in the different rehabilitation stages. At the same time, during the process of training and evaluation, doctors can monitor multiple patients in real time through computer analysis data, and adjust the training mode in real time, according to the actual situations of patients, to protect patients’ safety.

### 5.3. A Simulation Case

**Figure 15:**
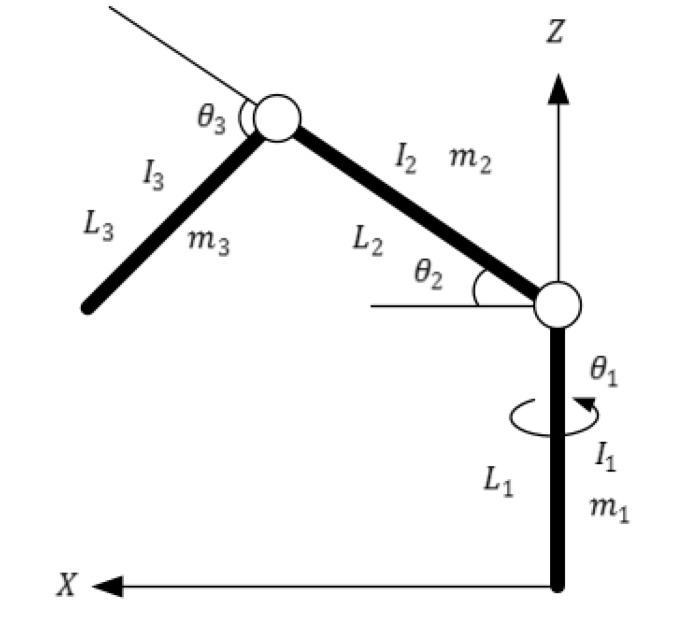
The sketch of a three DOF (Degree of Freedom) upper limb rehabilitation robot.

Figure 15 describes a three DOF (Degree of Freedom) upper limb rehabilitation robot. The dynamic model of this robot can be given as follows.

$$M\left(q,\sigma ,t\right)\ddot{q}+C\left(\dot{q},q,\sigma ,t\right)\dot{q}+G\left(q,\sigma ,t\right)=\tau \left(q,t\right)$$

with

$$M=\bar{M}+\Delta M$$

$$C=\bar{C}+\Delta C$$

$$G=\bar{G}+\Delta G$$

$$\bar{M}=\left[\begin{array}{ccc} m_{11} & m_{12} & m_{13}\\ m_{21} & m_{22} & m_{23}\\ m_{31} & m_{32} & m_{33} \end{array}\right]$$

$$m_{11}=I_{1}+a_{1}\cos^{2}\theta_{2}+a_{2}\cos^{2}(\theta_{2}+\theta_{3})+2a_{3}\cos\left(\theta_{2}\right)\cos(\theta_{2}+\theta_{3})$$

$$m_{12}=m_{21}=m_{13}=m_{31}=0$$

$$m_{22}=I_{2}+a_{1}+a_{2}+2a_{3}\cos\left(\theta_{3}\right)$$

$$m_{23}=m_{32}=a_{2}+a_{3}\cos\left(\theta_{3}\right)$$

$$m_{33}=I_{3}+a_{2}$$

$$\bar{C}=\left[\begin{array}{ccc} c_{11} & c_{12} & c_{13}\\ c_{21} & c_{22} & c_{23}\\ c_{31} & c_{32} & c_{33} \end{array}\right]$$

$$c_{11}=-\frac{1}{2}a_{1}{\dot{\theta }}_{2}\sin2\theta_{2}-\frac{1}{2}a_{2}({\dot{\theta }}_{2}+{\dot{\theta }}_{3})\sin\left(2\theta_{2}+2\theta_{3}\right)-a_{3}{\dot{\theta }}_{2}\sin\left(2\theta_{2}+\theta_{3}\right)-a_{3}{\dot{\theta }}_{3}\cos\left(\theta_{2}\right)\sin\left(\theta_{2}+\theta_{3}\right)$$

$$c_{12}=-\frac{1}{2}a_{1}{\dot{\theta }}_{2}\sin2\theta_{2}-\frac{1}{2}a_{2}{\dot{\theta }}_{1}\sin\left(2\theta_{2}+2\theta_{3}\right)-a_{3}{\dot{\theta }}_{1}\sin\left(2\theta_{2}+\theta_{3}\right)$$

$$c_{13}=-\frac{1}{2}a_{1}{\dot{\theta }}_{2}\sin\left(2\theta_{2}+2\theta_{3}\right)-a_{3}{\dot{\theta }}_{1}\cos\left(\theta_{2}\right)\sin\left(\theta_{2}+\theta_{3}\right)$$

$$c_{21}=-c_{12}$$

$$c_{22}=-a_{3}{\dot{\theta }}_{1}\sin\left(\theta_{3}\right)$$

$$c_{23}=-c_{13}$$

$$c_{32}=-a_{3}{\dot{\theta }}_{2}\sin\left(\theta_{3}\right)$$

$$c_{33}=0$$

$$\bar{G}=\left[\begin{array}{c} g_{1}\\ g_{2}\\ g_{3} \end{array}\right]$$

$$g_{1}=0$$

$$g_{2}=b_{1}\cos\left(\theta_{2}\right)+b_{2}\cos\left(\theta_{2}+\theta_{3}\right)$$

$$g_{3}=b_{2}\cos\left(\theta_{2}+\theta_{3}\right)$$

where $a_{1}=m_{2}r_{2}^{2}+m_{3}l_{2}^{2}$, $a_{3}=m_{3}r_{3}^{2}$, $a_{3}=m_{3}r_{3}l_{2}$, $b_{1}=\left(m_{2}r_{2}+m_{3}l_{2}\right)g$, $b_{2}=m_{3}r_{3}g$, $m_{i},\;i=1,\;2,\;3$ denotes the mass, $l_{i},\;i=1,\;2,\;3$ denotes the length, $I_{i},\;i=1,\;2,\;3$ denotes the moment of inertia, $\theta_{i},\;i=1,\;2,\;3$ denotes the angle.

Now we design the adaptive robust control based on the Udwadia-Kalaba theory proposed in Section 5.2 as:

$$\tau \left(\dot{q},q,t\right)=p_{1}\left(\dot{q,}q,t\right)+p_{2}\left(\dot{q},q,t\right)+p_{3}\left(\dot{q},q,t\right)$$

with

$$\begin{array}{cc} p_{1}\left(\dot{q},q,t\right)= & {\bar{M}}^{\frac{1}{2}}\left(q,t\right){\left(A\left(q,t\right){\bar{M}}^{-\frac{1}{2}}\left(q,t\right)\right)}^{+}[b\left(\dot{q,}q,t\right)\\ & +A\left(q,t\right){\bar{M}}^{-1}\left(q,t\right)(\bar{C}\left(\dot{q,}q,t\right)\dot{q}+G\left(q,t\right)-\lambda (A\left(q,t\right)\dot{q}\\ & -c\left(q,t\right)))] \end{array}$$

$$p_{2}\left(\dot{q,}q,t\right)=-k\bar{M}\left(q,t\right)A^{T}\left(q,t\right){\left(A\left(q,t\right)A^{T}\left(q,t\right)\right)}^{-1}P^{-1}\beta \left(\dot{q,}q,t\right)$$

$$p_{3}\left(\dot{q,}q,t\right)=-\bar{M}\left(q,t\right)A^{T}\left(q,t\right){\left(A\left(q,t\right)A^{T}\left(q,t\right)\right)}^{-1}P^{-1}\gamma \left(\hat{\alpha },\dot{q,}q,t\right)\mu \left(\hat{\alpha },\dot{q,}q,t\right)\Pi \left(\hat{\alpha },\dot{q,}q,t\right)$$

where

$$\gamma \left(\hat{\alpha },\dot{q,}q,t\right)=\{\begin{array}{ll} \frac{1}{\|\mu \left(\hat{\alpha },\dot{q,}q,t)\right\|} & \;\mathrm{if}\;\|\mu \left(\hat{\alpha },\dot{q,}q,t\right)\|>\hat{\epsilon }\;\\ \frac{1}{\hat{\epsilon }} & \;\mathrm{if}\;\|\mu \left(\hat{\alpha },\dot{q,}q,t\right)\|\leq \hat{\epsilon } \end{array}$$

$$\mu \left(\hat{\alpha },\dot{q,}q,t\right)=\beta \left(\dot{q,}q,t\right)\Pi \left(\hat{\alpha },\dot{q,}q,t\right)$$

$$\beta \left(\dot{q,}q,t\right)=A\left(q,t\right)\dot{q}-c\left(q,t\right)+\lambda \left(B\left(q,t\right)-d\left(q,t\right)\right)$$

$$\dot{\hat{\alpha }}=k_{1}\tilde{\Pi }\left(\dot{q,}q,t\right)\|\beta \left(\hat{\alpha },\dot{q,}q,t\right)\|-k_{2}\hat{\alpha }$$

The desired trajectory of each joint is given by the formulas below.

$$q_{d}\left(t\right)=\left[\begin{array}{c} 0.6\sin\left(\pi t\right)\\ 0.6\cos\left(\pi t\right)\\ 0.8\sin\left(\pi t\right) \end{array}\right]\mathrm{rad}$$

$${\dot{q}}_{d}\left(t\right)=\left[\begin{array}{c} 0.6\pi \cos\left(\pi t\right)\\ -0.6\pi \;\sin\left(\pi t\right)\\ 0.8\pi \;\cos\left(\pi t\right) \end{array}\right]\mathrm{rad}/s$$

$${\ddot{q}}_{d}\left(t\right)=\left[\begin{array}{c} -0.6\pi^{2}\sin\left(\pi t\right)\\ -0.6\pi^{2}\;\cos\left(\pi t\right)\\ -0.8\pi^{2}\;\sin\left(\pi t\right) \end{array}\right]\mathrm{rad}/s^{2}$$

**Figure 16:**
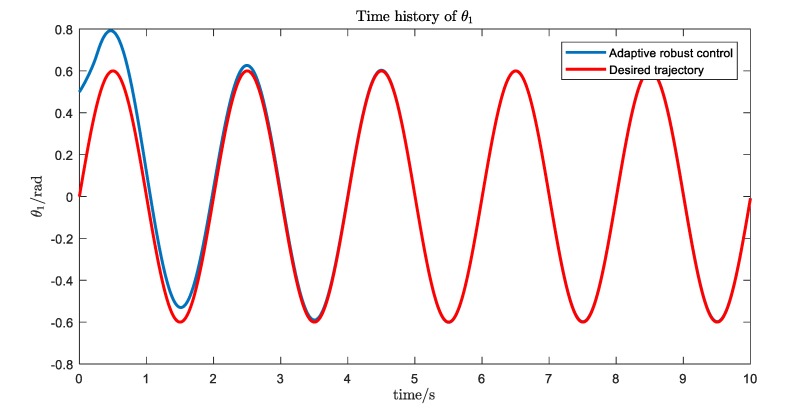
The trajectory tracking of joint 1.

**Figure 17:**
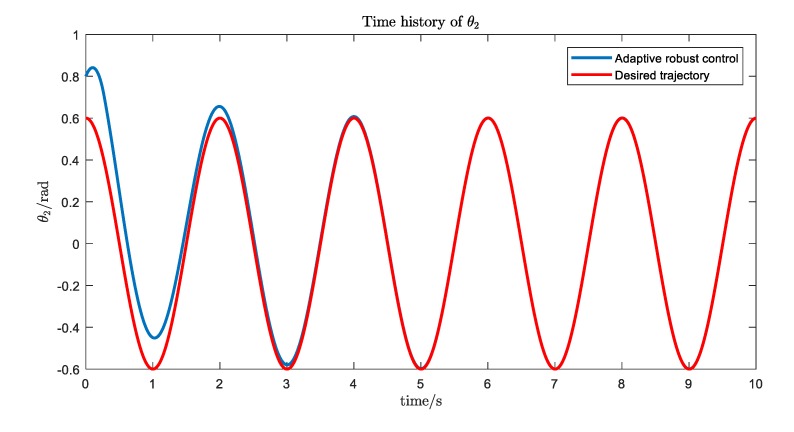
The trajectory tracking of joint 2.

**Figure 18:**
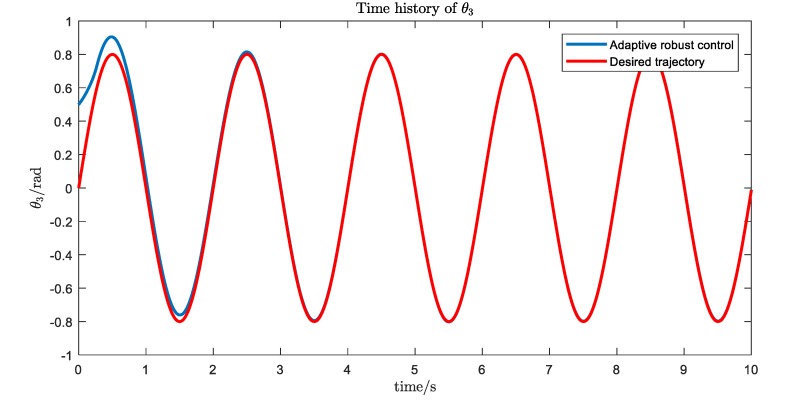
The trajectory tracking of joint 3.

**Figure 19:**
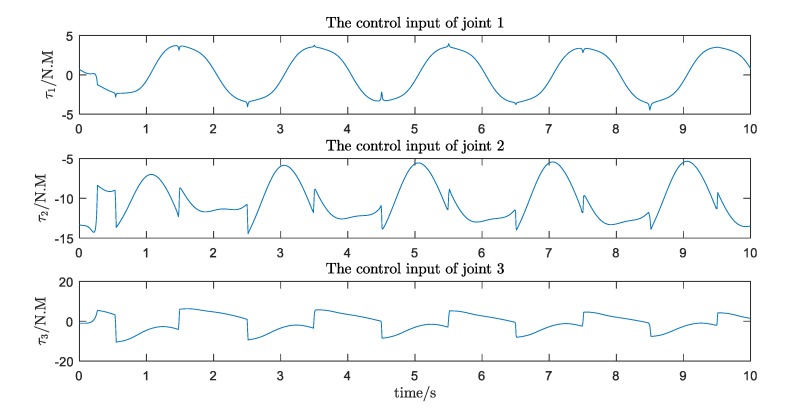
The control input of each joint.

The simulation results are shown in Figure 16, Figure 17, Figure 18 and Figure 19. We can see that, with the proposed Udwdia-Kalaba theory based on adaptive robust control, the actual trajectory of each joint can track the desired requirements.

## 6. Conclusions

To better heal upper-limb injuries and improve the ability of doctors to optimize both medical solutions and the management of medical institutions, this paper studied upper limb rehabilitation instruments, based on the research and development of existing instruments and their problems. The main focus was to design a natural interaction and collaboration-intelligent upper limb rehabilitation training robot. The designed robot’s innovations and effect are as follows.

(1) A new structure for an upper limb rehabilitation robot is proposed. It combines a flexible rope with an exoskeleton in order to ensure that the moving parts connected with the patient’s upper limbs are light, accurate, and flexible.
(2) Applying the theories and technologies of ergonomics, virtual reality, information fusion, big-data analysis, and deep learning, a collaborative, efficient, and intelligent remote rehabilitation system based on a human’s natural response is constructed.
(3) For this multi-degree-of-freedom robot system, the Udwadia-Kalaba approach is applied to establish the dynamic equation of the system. Based on this explicit dynamic equation, optimal adaptive robust control design with fuzzy set theory is presented for the motion control of the system.
(4) The new design will help improve the interest of patients in participating in the training and improve the effects of the rehabilitation, help doctors design a good rehabilitation training program, and help hospitals manage patients in real time and summarize the experiences.

Our future work will mainly focus on the experimental verification. A large number of case trainings will be done to verify the flexibility, comfort, and safety of the system.

## Figures and Tables

**Table 1:** Comparison of robotic devices for upper limb rehabilitation.

System Name	DOF (Degree of Freedom)	Supported Movements	Type, Field of Application
ACRE, Schoone [41]	5	Shoulder * elbow	Stationary system (end-effector-based), physical therapy
MariBot, Rosati [42]	5	Shoulder * elbow	Stationary system (end-effector-based, cable-driven robot), physical therapy
Robotherapist, Furusho [43]	6	Shoulder * elbow * forearm * wrist	Stationary system (end-effector-based), physical therapy
iPAM, Culmer [44]	6	Shoulder * elbow * forearm	Stationary system (2 robotic arms), physical therapy
The proposed rehabilitation robot	7	Shoulder * elbow * wrist	Stationary system (end-effector-based), physical therapy and assessment of therapy results

* means and.
